# Supplementary material for: Accounting for spatial habitat and management boundaries when estimating forest bird population distribution and density: inferences from a soap film smoother
Source: PeerJ. 2023 Jun 14;11:e15558. doi: 10.7717/peerj.15558 (PMC10276562; doi:10.7717/peerj.15558)
Supplement: Supplemental Information 1 [file peerj-11-15558-s001.pdf]

SUPPLEMENTAL MATERIALS

(available online as Supporting Online Information Appendix at <URL>).
Camp, R.J., D.L. Miller, S.T. Buckland, and S.J. Kendall. Soap film smoothers to estimate bird abundances.
The findings and conclusions in this article are those of the author(s) and do not necessarily represent the views of the U.S. Fish and Wildlife Service. Any use of trade, firm, or product names is for descriptive purposes only and does not imply endorsement by the U.S. Government.

Supporting Online Information Appendix A

In a preliminary analysis we selected among Poisson, negative binomial and Tweedie distributions to model the response distribution. These analyses were performed on a TPRS smooth and the selected distribution was applied to the soap film smoother. We restricted the Tweedie distribution power parameter to $p = (1.1, 2)$ following recommendations provided in the `mgcv`, version 1.8-17, package help pages and used a method of bisection to approximately identify the likelihood maximum. Sensitivity to the choice of response distribution and model assumptions were checked through inspection of the deviance residuals following approaches suggested by Wood (2017). Akaike's information criterion (AIC) was used to select among distributions.

We chose the negative binomial distribution with log-link for modelling the 2002 'Ākepa spatial patterns. This model provided a reasonably good fit to the residuals (Supporting Online Information Appendix A Figs. 1–3). The AIC value for the negative-binomial distributed model was about 2 AIC units larger than the Poisson distributed model (Supporting Online Information Appendix A Table 1). Thus, AIC alone could not be used to select between these two models (Burnham and Anderson, 2002). AIC statistics clearly eliminated the Tweedie distribution (Supporting Online Information Appendix A Table 1). QQ-plots showed the negative binomial distribution did a better job of following the identity line from the smallest through middle values than the other two models (Supporting Online Information Appendix A Fig. 1). All three models deviated at the largest values. Therefore, we based our model selection on the QQ-plots.

Diagnostic plots for the negative binomial distribution fitted with soap film model formulation to the 2002 'Ākepa data indicated the model adequately fit the data. Inspection of residual diagnostic plots appeared reasonable with acceptable behaviour for the deviance residuals and error distribution (Supporting Online Information Appendix A Figs. 2 and 3). The diagnostics of the soap film residuals are very similar to those of the TPRS model where the QQ-plot is very close to the straight line indicating that the distribution is reasonable (Supporting Online Information Appendix A Fig. 2, top left panel), the residuals versus linear predictor values appears to be reasonable with a strong banding pattern (Supporting Online Information Appendix A Fig. 2, top right panel), the histogram of residuals approximates normality with a spike at zero (Supporting Online Information Appendix A Fig. 2, bottom left panel), and the diagonal pattern in the response versus fitted values draws into question the assumption of constant variance (Supporting Online Information Appendix A Fig. 2, bottom right panel). Points from the sorted deviance residuals seem to fall about the straight line and well within the simulated theoretical quantiles band of grey lines, which provides evidence the numbers came for the theoretical distribution (Supporting Online Information Appendix A Fig. 3). Effect plots for the soap film model terms are shown in Supporting Online Information Appendix A Fig. 4, while a description of the effects is presented in the text.

Diagnostic plots for the negative binomial distribution fitted with the TPRS model formulation to the

Supporting Online Information Appendix A Table 1. Model selection statistics for the Poisson, negative binomial and Tweedie distributions. Presented are the smoother log-likelihood (logLik), effective degrees of freedom (EDF), Akaike's information criterion (AIC), and Δ AIC.

Model	logLik	EDF	AIC	Δ AIC
Poisson	-275.961	20.754	593.432	0
negative binomial	-276.677	21.107	595.569	2.137
Tweedie	-293.101	20.920	628.043	34.611

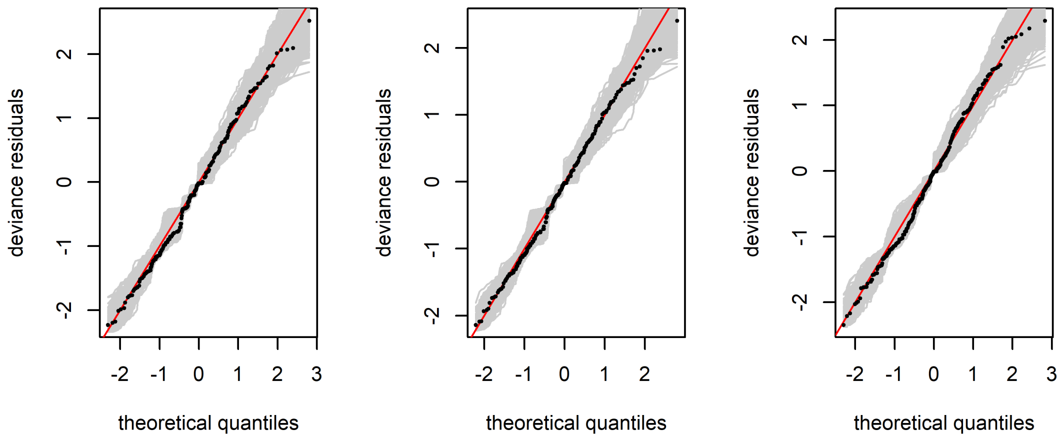

Supporting Online Information Appendix A Figure 1. QQ plots of sorted deviance residuals (black dots) for the spatial GAM against theoretical quantiles (grey lines; 1,000 replicates) fitted with the Poisson (left panel), negative binomial (middle panel) and Tweedie (right panel) distributions to the 2002 'Ākepa *Loxops coccineus* count data from a TPRS model.

Supporting Online Information Appendix A Table 2. Effective degrees of freedom (EDF), reference degrees of freedom (rf), and basis complexity (*k*-index) for each term in the TPRS smooth spatial model.

Term	EDF	rf	*k*-index
`s(Easting, Northing)`	18.59	126	1.02

2002 'Ākepa data (Supporting Online Information Appendix A Figs. 1 and 5). The QQ-plot is very close to the straight line indicating that the distribution is reasonable (Supporting Online Information Appendix A Fig. 5, top left panel), the residuals versus linear predictor values appears to be reasonable, although strong banding patterns are obvious (Supporting Online Information Appendix A Fig. 5, top right panel), the histogram of residuals approximates normality with a spike at zero (Supporting Online Information Appendix A Fig. 5, bottom left panel), and the diagonal pattern in the response versus fitted values reveals that the assumption of constant variance is questionable, again with a strong banding pattern (Supporting Online Information Appendix A Fig. 5, bottom right panel). Points from the sorted deviance residuals seem to fall about the straight line and well within the simulated theoretical quantiles band of grey lines, which provides evidence the numbers came for the theoretical distribution (Supporting Online Information Appendix A Fig. 6).

Effect plots for the TPRS model term is shown in Supporting Online Information Appendix A Fig. 7. The EDF on the `Easting` and `Northing` smooth term was greater than zero, significant and the function was nonlinear (Supporting Online Information Appendix A Table 2; Supporting Online Information Appendix A Fig. 7).

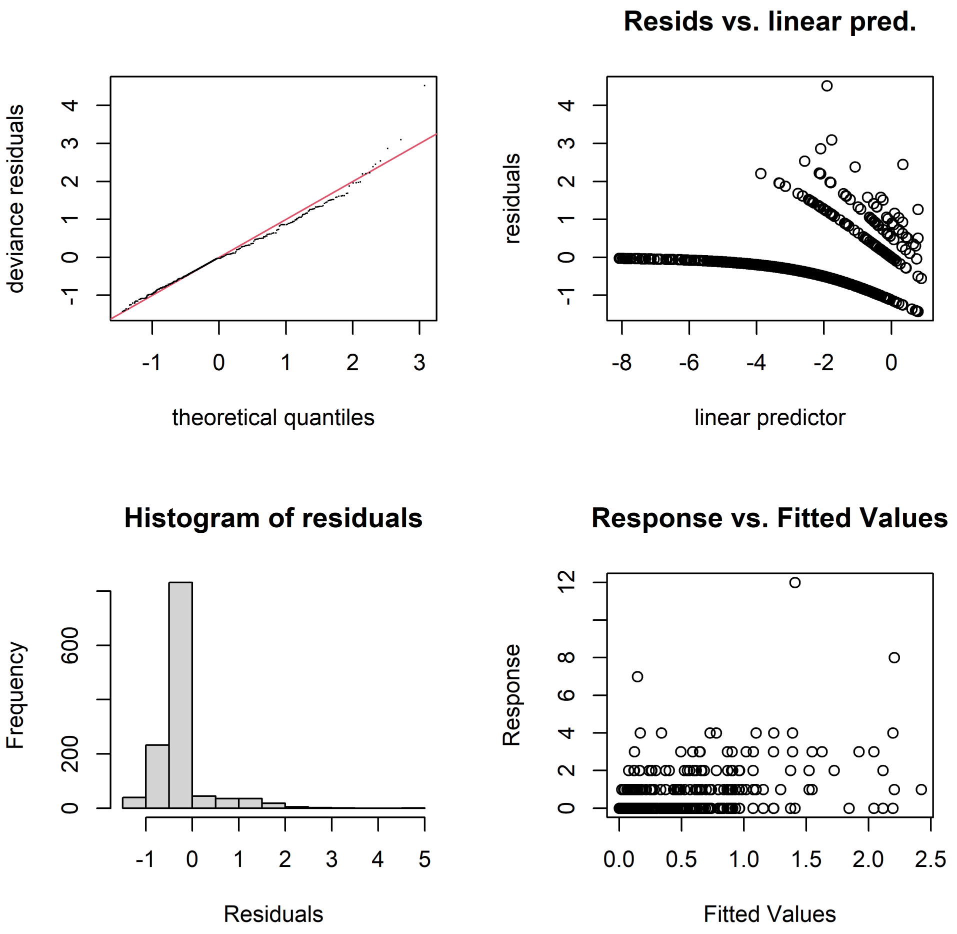

Supporting Online Information Appendix A Figure 2. Diagnostic plots of individual parameters for spatial GAM with a negative binomial distribution fitted with soap film model formulation to the 'Ākepa *Loxops coccineus* count data for the 2002 survey. Diagnostic are QQ-plot (top left panel), residuals versus linear predictor (top right panel), histogram of residuals (bottom left panel), and response versus fitted values (bottom right panel).

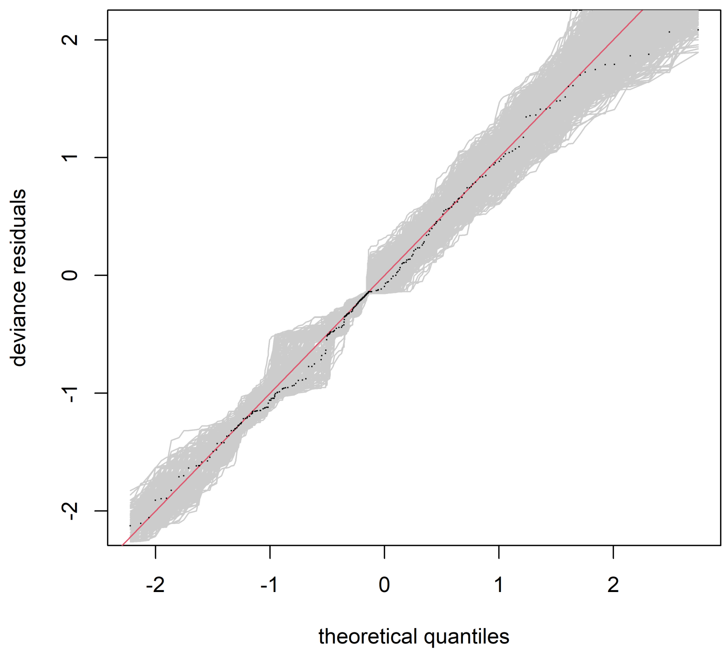

Supporting Online Information Appendix A Figure 3. Sorted deviance residuals (black line) for the spatial GAM against theoretical quantiles (grey lines; 1,000 replicates) fitted to the 2002 'Ākepa *Loxops coccineus* count data for the negative binomial distribution with the soap film model formulation.

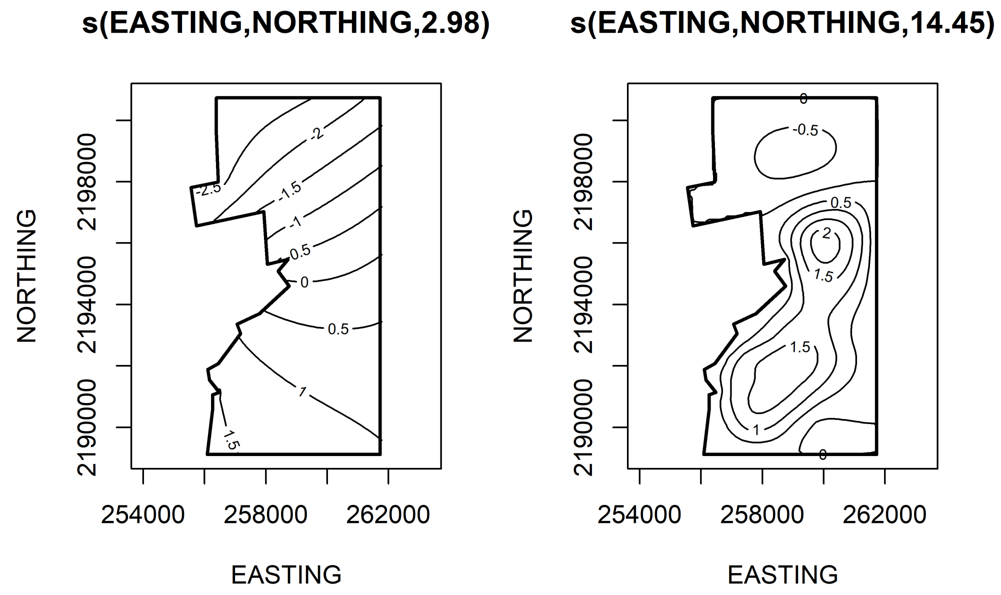

Supporting Online Information Appendix A Figure 4. Estimated model terms for the spatial soap film fitted to the 'Ākepa *Loxops coccineus* count data. The 2D contour plots represent 0.5 unit change are shown as blue lines and the EDF is provided in the plot panel title. Estimates provided on the scale of the link function.

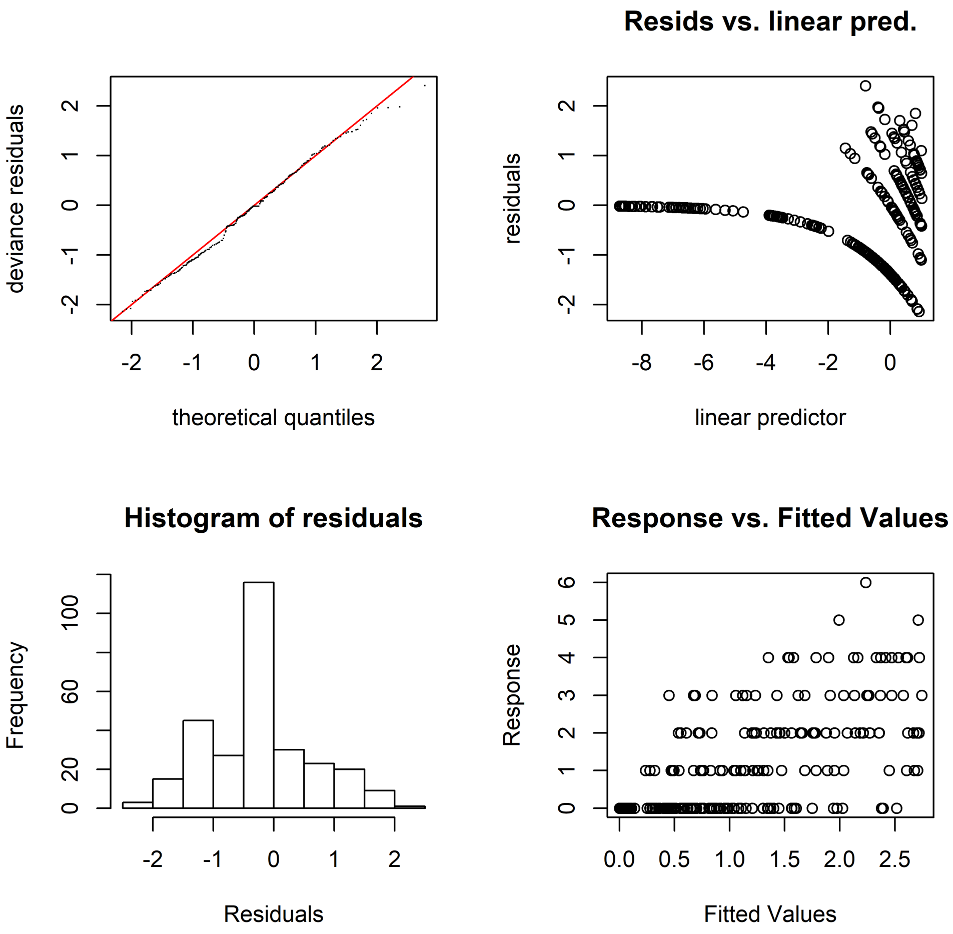

Supporting Online Information Appendix A Figure 5. Diagnostic plots of individual parameters for spatial GAM with a negative binomial distribution fitted with the TPRS model formulation to the ʻākepa *Loxops coccineus* count data for the 2002 survey. Diagnostic are QQ-plot (top left panel), residuals versus linear predictor (top right panel), histogram of residuals (bottom left panel), and response versus fitted values (bottom right panel).

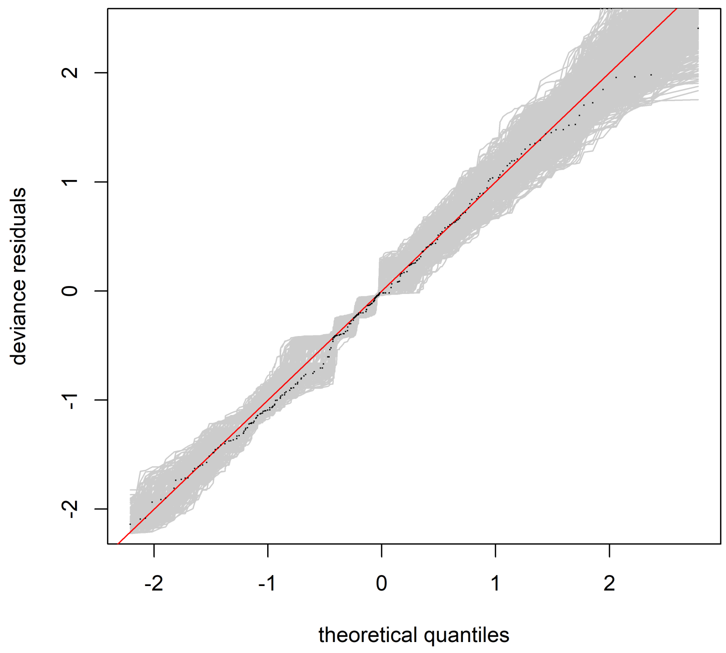

Supporting Online Information Appendix A Figure 6. Sorted deviance residuals (black line) for the spatial GAM against theoretical quantiles (grey lines; 1,000 replicates) fitted to the 2002 'Ākepa *Loxops coccineus* count data for the negative binomial distribution with the TPRS model formulation.

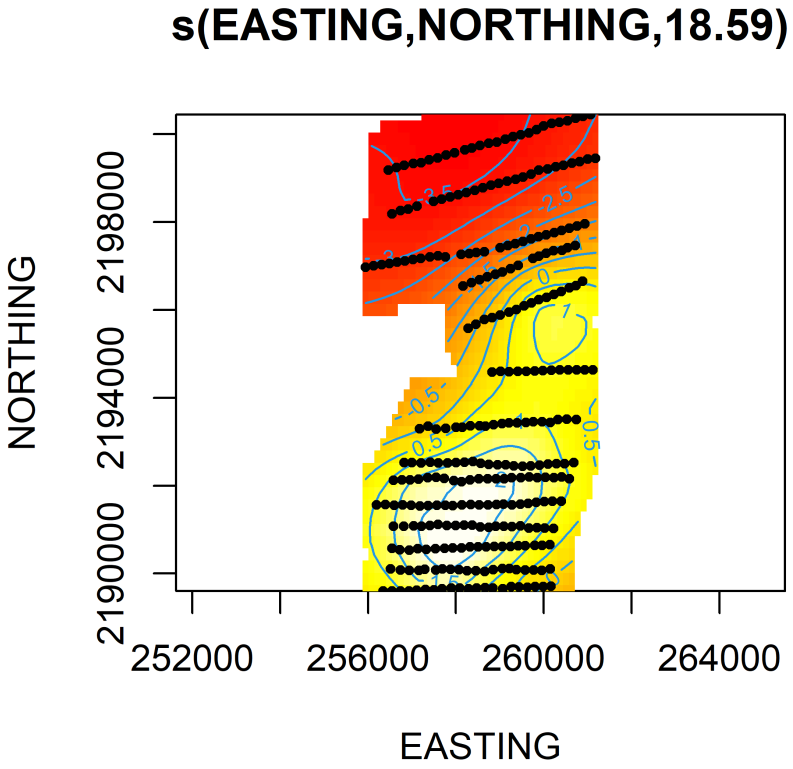

Supporting Online Information Appendix A Figure 7. Estimated model terms for the spatial TPRS fitted to the 'Ākepa *Loxops coccineus* count data. The locations of the points are plotted as black dots on the 2D contour plots and the EDF is provided in the plot panel title. Contours represent 0.5 unit change and are shown as blue lines. Estimates provided on the scale of the link function.

Supporting Online Information Appendix B

R code for spatial soap film model fitted to counts with an offset accounting for detectability while controlling the boundary behaviour to produce a density surface. We specified the basis in two parts to ensure adequate complexity for both the boundary and interior smoother components.

```
# Load domain boundary, which is a list of two columns giving the
# coordinates of points defining the boundary
bnd <- read.csv(filename, header=TRUE, fill=TRUE)

# Define the location of interior knots for the smooth
# set a regular grid with locations every 730m east and 670m north
# across the study area; this ensured that all knots are
# either inside or outside the boundary
gp <- expand.grid(EASTING=seq(from=255400, to=261500, by=730),
                  NORTHING=seq(from=2189000, to=2200800, by=670))
names(bound[[1]]) <- c("EASTING", "NORTHING", "f")
knots <- gp[with(gp, inSide(bound, EASTING, NORTHING)), ]

# Soap film model
SF <- gam(Count ~ s(EASTING, NORTHING, k=20, bs="sf",
                    xt=list(bnd=bnd, nmax=nmax)) +
                  s(EASTING, NORTHING, k=20, bs="sw",
                    xt=list(bnd=bnd, nmax=nmax)) +
                  offset(log(nu)),
                  family=nb(),
                  data=data,
                  method="REML",
                  knots=knots)
```

The effective area searched, ν, was calculated as the truncation distance squared times π times the detection probability. We specified the boundary and interior basis arguments for the soap film smooth separately. An alternative formulation is to use the `bs="so"` argument. This construct is a wrapper for the boundary and interior smooths. While the `bs="so"` construct is easier to use, it does not allow for checking that sufficiently large basis complexity has been selected for each soap film component.

Supporting Online Information Appendix C

Hazard-rate detection function diagnostic plots indicated that the model adequately fit the data (Supporting Online Information Appendix C Fig. 1).

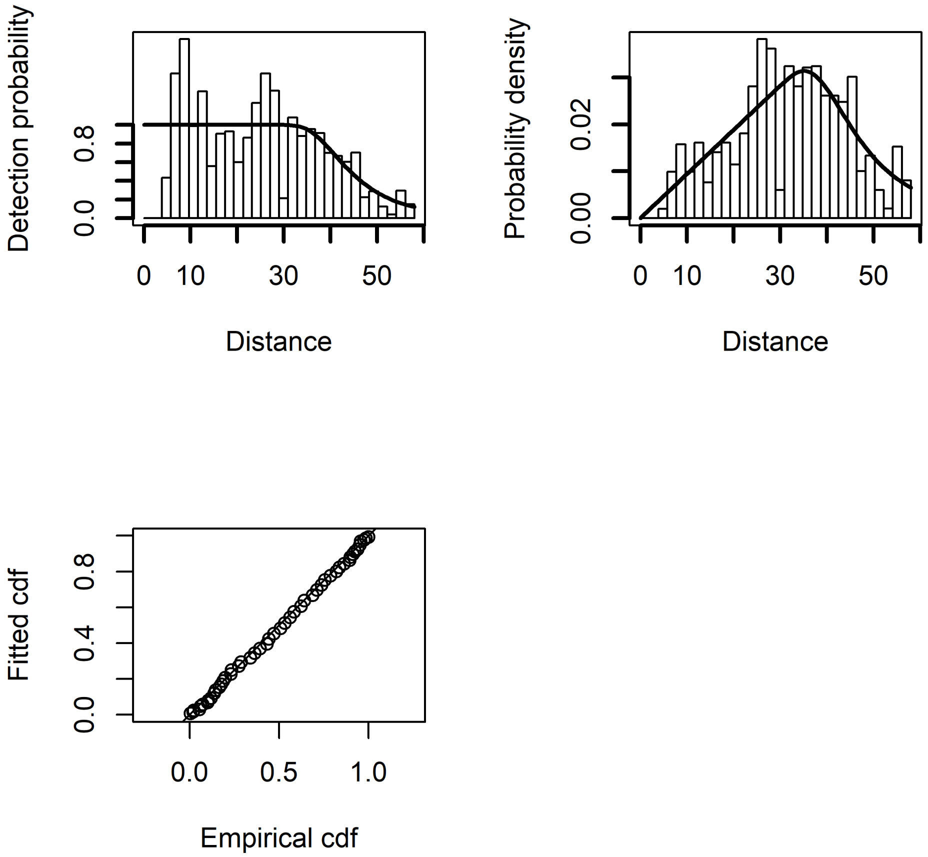

Supporting Online Information Appendix C Figure 1. Detection function plots for the hazard-rate model without series expansion or covariates fitted to the 2002 'Ākepa *Loxops coccineus* detections in the forest stratum. Plots represent the average detection probability (left top panel), probability density (right top panel) and QQ-plot (bottom panel). There is moderate deviation in the histogram in the probability plots and the points seem to fall about the straight line of the QQ-plot, which provides evidence the function adequately fits the data.
